# Impact of an Expansion of a Clinical Nutrition Curriculum on Pre-Clerkship Medical Students’ Perception of Their Knowledge and Skills Related to Performing a Nutritional Assessment

**DOI:** 10.3390/nu13114081

**Published:** 2021-11-15

**Authors:** Trey Keel, Doreen M. Olvet, Marie Cavuoto Petrizzo, Janice T. John, Rebecca Dougherty, Eva M. Sheridan

**Affiliations:** Department of Science Education, Donald and Barbara Zucker School of Medicine at Hofstra/Northwell, Hempstead, NY 11549, USA; TKeel@northwell.edu (T.K.); Marie.C.Petrizzo@hofstra.edu (M.C.P.); Janice.T.John@hofstra.edu (J.T.J.); rebecca.h.dougherty@hofstra.edu (R.D.); Eva.M.Sheridan@hofstra.edu (E.M.S.)

**Keywords:** nutrition, nutrition education, obesity, counseling, undergraduate medical education

## Abstract

Learning how to provide nutritional counseling to patients should start early in undergraduate medical education to improve the knowledge, comfort, and confidence of physicians. Two nutrition workshops were developed for first-year medical students. The first workshop, co-led by physicians and registered dieticians, focused on obtaining nutrition assessments. The second workshop focused on the appropriate dietary counseling of patients with chronic kidney disease and cardiovascular risk. We surveyed students before workshop 1, after workshop 1, and after workshop 2 to assess their perceptions of the value of physician nutrition knowledge and counseling skills as well as their own comfort in the area of nutritional knowledge, assessment, and counseling. We found a significant improvement in their self-assessed level of knowledge regarding counseling patients, in their comfort in completing a nutritional assessment, and in their confidence in advising a patient about nutrition by the end of the first workshop. By the time of the second workshop five months later, students continued to report a high level of knowledge, comfort, and confidence. The implementation of clinical nutrition workshops with a focus on assessment, management, and counseling was found to be effective in increasing student’s self-assessed level of knowledge as well as their confidence and comfort in advising patients on nutrition. Our findings further support the previous assertion that clinical nutrition education can be successfully integrated into the pre-clerkship medical school curriculum.

## 1. Introduction

Despite the burden that obesity places on US public health and US national medical expenditures, over 90% of physicians do not council their patients about weight loss [1,2]. There are many reasons for this, including insecurity in nutrition counseling and gaps in nutrition knowledge [2,3]. Nutrition habits develop early in life and being obese as a child leads to increased risk of obesity and metabolic syndrome in adulthood [4,5]. Ultimately, today’s overweight children will become adults with chronic conditions requiring treatment by our future physicians [6]. The need for clinical nutrition education is only increasing and educational reform is required to tackle this growing epidemic. 

Learning how to counsel patients on nutrition and obesity management should start as early as in undergraduate medical education (UME). However, both the time and resources allotted in UME are insufficient to provide medical students with adequate training in the area of clinical nutrition education [7,8,9]. The nutrition education that is currently provided is largely focused on basic science and lectures, and learners report rarely having the opportunity to practice or observe physicians demonstrating best practices in nutrition counseling in a clinical setting [8,9]. As a result, medical students do not feel adequately prepared and lack the confidence to incorporate nutrition counseling into their practice [8]. Insecurity in counseling extends into graduate medical education as well. For example, pediatric residents have reported not being satisfied with their nutrition knowledge and not feeling comfortable or able to effectively counsel their patients or parents on the matter of nutrition [10]. 

Incorporating new and more robust nutrition education strategies into UME classroom instruction could be beneficial to future physicians. Reforming nutrition education, such as incorporating culinary medicine courses into UME curricula that offer a hands-on approach to learning nutrition, has been effective in increasing students’ nutritional knowledge and their confidence in providing nutrition and obesity counseling [11]. Other methods of applied learning, such as practical nutrition electives and diet behavior electives, have also shown success at various US medical schools [12]. Our previous work demonstrated that having students apply their knowledge of nutrition in case-based scenarios led to an improvement in their perceived knowledge, comfort, and confidence in providing nutrition counseling to patients [12].

To expand on this initial success and further bolster our nutrition curriculum, a second nutrition session was added to our curriculum to allow students to practice clinical skills. Students utilize role-play with faculty members and practice recording nutrition history in this practical session. This model reflects Kolb’s experiential learning theory, in which information is grasped by learners (i.e., abstract conceptualization in the first session) and transformed into practice (i.e., active experimentation, or role play, in the second session) [13]. We assessed students’ confidence in the area of nutritional knowledge, assessment, and counseling based on the continuum of our existing nutrition session and provided an additional novel nutrition session for preclinical medical students. We predicted that adding a second practical skills session to the curriculum would further improve students’ perceived knowledge, comfort, and confidence in taking a patient’s nutrition history. 

## 2. Materials and Methods

### 2.1. Participants

Two nutrition workshops were provided as mandatory curricular sessions for the entire cohort of 102 students matriculated in their first year of medical school (MS1) at the Donald and Barbara Zucker School of Medicine at Hofstra/Northwell (ZSOM) in the 2020–2021 academic year.

### 2.2. Procedure

Workshop 1: We held the first nutrition workshop during a course titled “Continuity and Change: Fueling the Body (FTB)”, which is a required course that covers the gastrointestinal system, including metabolism and the processing of nutrients for digestion and absorption. Students were given pre-workshop readings to enhance their basic understanding of how to obtain a nutrition history, inform them about nutrition myths, teach them how to implement healthy dietary practices, improve their understanding of common dietary patterns, and help them to appreciate the value of various patient nutrition handouts. 

The first workshop was structured as a 45 min large group session on nutrition basics, followed by a 55 min interactive small group session led by faculty members. The large group framing session was led by a registered dietician and a physician. The concepts covered in the large group session included the clinical significance and elements of a nutrition assessment, the importance of cultural humility when discussing nutrition, the discussion of dietary interventions in the clinical setting, and the role of an interprofessional relationship with registered dieticians. The large group also included a hands-on education segment focused on reading and critically appraising a nutrition label. 

The large group was then broken up into small groups of 9–10 students, with a registered dietitian and physician leading the discussions. Most of the small group sessions focused on practicing the clinical skill of obtaining a basic nutrition assessment using 2 cases for role play. Students practiced taking a nutrition history while faculty members played the role of the patient. The first case depicted a 40-year-old patient with chronic fatigue, morbid obesity, and progressive weight gain. The second case depicted a 78-year-old patient with chronic gastroesophageal reflux disease, 20-pound weight loss, and malnutrition. The registered dietitian and physician then facilitated a discussion of the patients’ nutrition problems and discussed intervenable areas with the students. Each case discussion lasted 20 min. 

Workshop 2: The second nutrition workshop was held 5 months later during the MS1 Homeostasis (HOM) course. This course highlighted the interrelationships of the cardiac, renal, and pulmonary systems from the cellular level to clinical manifestations. The goals of this nutrition workshop were to describe the components of cardiac and renal healthy diets, review the evidence behind diets that are commonly prescribed for patients with renal and cardiovascular disease, and have students practice advising a patient on dietary modifications for managing chronic kidney disease and cardiovascular risk. Prior to the nutrition session, students were assigned pre-readings on evidence-based diets for cardiac and renal health as well as patient handouts on popular diets such as Dietary Approaches to Stop Hypertension (DASH) [14], the Mediterranean diet, and whole-foods plant-based diets.

This workshop was structured as a 70 min interactive experience. Due to the COVID-19 pandemic, it was converted into a virtual workshop using Zoom video conferencing (Zoom Video Communications, San Jose, CA, USA). The first 55 min were spent in an interactive large group session. A cardiologist led the first half of the large group session, while the second half was led by a nephrologist. This session was framed as a continuation of the first nutrition workshop in which students would build upon the base nutrition education knowledge they had already learned. Faculty discussed the association between dietary practices and patterns and the development and management of chronic cardiac and renal diseases, including the basic principles of popular diets. Faculty members reviewed dietary approaches to prevent and manage both cardiac and renal diseases and modeled, through role play, dietary counseling using the Ask, Respond, Tell (ART) framework and the teach back method [15]. ‘Ask’ refers to the teacher asking the learner what they understand about the topic. ‘Respond’ refers to the teacher responding to that knowledge with active listening and/or empathy. ‘Tell’ refers to the teacher offering up their perspective and knowledge on the learning topic.

Following the large group session, students were randomly paired in Zoom breakout rooms and were provided with a clinical vignette and a checklist to self-evaluate their communication skills. The vignette depicted a 51-year-old patient with progressively worsening polycystic kidney disease and clinical manifestations of fluid overload. They were tasked to take turns role-playing counselling the patient about their diet. Student A was assigned the role of the physician and was tasked with obtaining a dietary history and counselling the patient (Student B) regarding their salt and water intake. The students then switched roles and Student B was advised to interpret abnormal laboratory results for the patient (Student A) and counsel the patient about their dietary potassium and phosphorus intake. 

### 2.3. Evaluation

The survey was distributed through Qualtrics (https://www.qualtrics.com/ accessed on 15 January 2021) to all MS1s before the nutrition workshop (pre-workshop 1), after the nutrition workshop (post-workshop 1), and after a second nutrition workshop that took place five months later (post-workshop 2). Each survey was comprised of six elements designed to assess the students’ perceptions of the value of physician nutrition knowledge and counseling skills as well as their own comfort with nutritional knowledge, assessment, and counseling. Reponses were based on a 5-point Likert scale: 1 = strongly disagree, 2 = disagree, 3 = neither agree nor disagree, 4 = agree, and 5 = strongly agree.

### 2.4. Statistical Analysis

Data were statistically evaluated using IBM SPSS Statistics (SPSS Inc., Chicago, IL, USA, Version 24.0). Collected survey data were consolidated into three categories: strongly disagree/disagree, neither agree nor disagree, and strongly agree/agree. Data were presented as the percentage of students who responded in each of the categories for the six survey items at each of the time-points: pre-workshop 1, post-workshop 1, and post-workshop 2. The Wilcoxon Signed Rank test (Z statistic) was used to determine differences in perception before and after the nutrition session, while the Friedman test (χ^2^ statistic) was used to determine differences across all three time-points (pre-test vs. post-test vs. follow-up). Significant results from the Friedman tests were followed up with post hoc Wilcoxon Signed Rank tests. A *p* value ≤ 0.05 was considered to be statistically significant. Hofstra University’s Institutional Review Board (IRB) approved this research under the exempt review procedures. 

## 3. Results

One hundred and two first-year medical students (100%) participated in the nutrition workshops. Survey data for both pre- and post-workshop 1 were matched for 51 students (50%) and matched data for all three time-points were available for 30 students (29%). The following data were not included in the current analysis: 28 students participated in only the pre-workshop 1 survey, 14 participants only had data for the pre-workshop 1 and post-workshop 2 surveys, and 9 students did not participate in any of the surveys. 

### 3.1. Pre- and Post-Workshop 1 Survey Data

**Figure 1:**
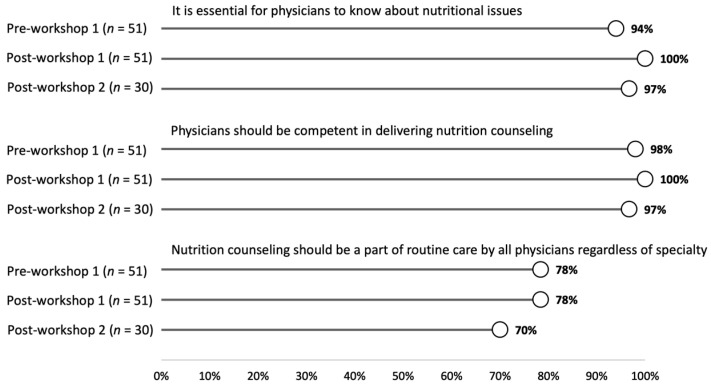
The percent of students who responded strongly agree or agree to each question at the pre-workshop 1, post-workshop 1, and post-workshop 2 time-points.

Figure 1 shows the percent of students who strongly agreed/agreed to the six survey items at the pre- and post-workshop 1 time-points for the 51 students (50% of the cohort) who responded to both surveys. There was no significant change in student perception regarding the knowledge and skills physicians should possess about nutrition counseling between the pre- and post-workshop timepoints. An overwhelming majority of students strongly agreed/agreed that physicians must know about nutritional issues (94% pre- and 100% post-workshop 1; *Z* = −1.6, *p* = 0.10), should be competent in providing nutrition counseling (98% pre- and 100% post-workshop 1; *Z* = −1.0, *p* = 0.32,) and that nutrition counseling should take place as part of routine care by all physicians (78% pre- and 78% post-workshop 1; *Z* = 0, *p* = 1.0). 

**Figure 2:**
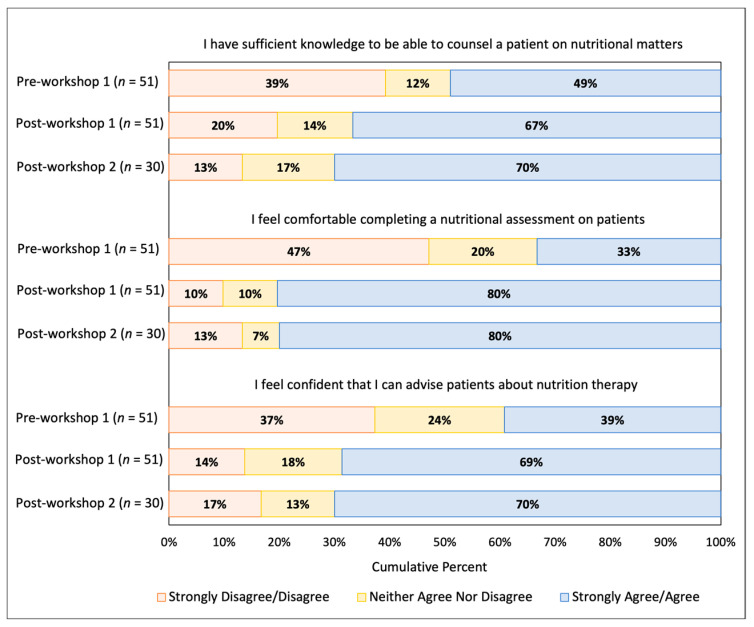
Cumulative percent of student responses for each question at the pre-workshop 1, post-workshop 1, and post-workshop 2 time-points.

Figure 2 shows the cumulative percentage of student responses regarding the students’ self-perceived knowledge, comfort, and confidence before and after the nutritional workshop. We found a significant improvement in all three items after the workshop. Sixty-seven percent of students (*N* = 34) strongly agree/agreed that they had sufficient knowledge to provide nutrition counseling to a patient after the workshop compared to 49% pre-workshop (*N* = 25; *Z* = −2.3, *p* = 0.02). Eighty percent of students (*N* = 41) strongly agree/agreed that they were comfortable in administering a nutritional assessment after the workshop compared to 33% who were comfortable pre-workshop (*N* = 17; *Z* = −4.6, *p* < 0.001). Finally, 69% (*N* = 35) of the students strongly agree/agreed that they felt confident that they could advise patients about nutrition after the workshop compared to 39% pre-workshop (*N* = 20; *Z* = −3.5, *p* < 0.001).

### 3.2. Long Term Follow-Up Survey Data

Survey data for all three time-points were available for a subset of 30 students (29% of the cohort) who responded to all three surveys. Data from the post-workshop 2 survey are included in Figure 1 and Figure 2. Students continued to strongly agree/agree that physicians should know about nutritional issues (97% at post-workshop 2; (χ^2^(2) = 1.0, *p* = 0.61), that physicians should be capable of delivering nutrition counseling with no significant change after workshop 2 (97% at post-workshop 2; (χ^2^(2) = 2.0, *p* = 0.37), and that nutrition counseling should be part of routine care by all physicians (70% at post-workshop 2; χ^2^(2) = 2.0, *p* = 0.37). There was also no significant change in the students’ perceived knowledge (post-workshop 1 vs. post-workshop 2; *Z* = −1.3, *p* = 0.19), comfort in completing a nutritional assessment (post-workshop 1 vs. post-workshop 2; *Z* = −0.09, *p* = 0.93), or confidence in advising patients after the second workshop (post-workshop 1 vs. post-workshop 2; *Z* = −0.75, *p* = 0.45).

## 4. Discussion

Survey data indicated that the first nutrition workshop resulted in an overall improvement in self-assessed knowledge, comfort, and confidence. Prior to attending the nutrition workshop, only half of our medical students felt that they had sufficient medical knowledge to counsel a patient on nutritional matters, and only a third felt comfortable in completing a nutritional assessment. After the first workshop, students reported that they had adequate knowledge to counsel a patient on nutrition matters, felt comfortable completing a nutrition assessment, and felt confident advising patients about nutrition, which is consistent with our previous findings [12].

The second nutrition session reinforced students’ perception that they possessed sufficient knowledge to counsel patients on nutrition and enabled students to retain the confidence they had gained from the first session in completing a nutritional assessment and advising patients on nutrition. Based on survey data from a subset of students (29%),they consistently agreed that nutrition knowledge and competency in nutrition counseling are essential for physicians, and that nutrition counseling should be part of the routine care offered by all physicians. It has been reported that students’ attitudes regarding nutrition education are influenced by clinically relevant teaching and reinforcing the importance of incorporating clinical nutrition early on in the medical school curriculum [16]. The interprofessional nature of the session with registered dieticians may have helped the students to understand their role in nutrition counseling as physicians and shown them how to effectively collaborate with registered dieticians in the future [16].

It has been reported that healthcare providers lack confidence in their knowledge of nutrition and/or ability to council patients [17,18,19]. The findings from this study further highlight the impact that short, interactive nutrition sessions have on medical students’ confidence in providing nutrition counseling. Enhancing confidence is crucial, as it breaks down the insecurity barrier preventing students counseling patients on nutrition and increases their self-efficacy. The design of our workshop was structured similarly to a previous nutrition workshop carried out at ZSOM and aligns with the American Nutrition Association’s personalized nutrition model [12,20]. Our workshops helped to integrate the basic science and clinical concepts taught in the existing curriculum using realistic and common patient presentations as the context for learning nutrition concepts and practicing important skills. 

There are some limitations to our study that warrant attention. This workshop was carried out at a single institution; therefore, further research at additional medical schools will be needed to confirm the generalizability of these findings. Another limitation of the study was the low response rate of the students who completed the surveys (50% for the first two surveys and 29% for all three surveys). There may have been some selection bias in the students who chose to complete all surveys based on their personal interest in nutrition, and therefore the effectiveness of the interventions may not be applicable to all medical students. The survey we used was not validated; however, the items were carefully developed to align with the learning objectives of the workshop. Finally, there was a lack of objective data regarding the effects of the nutrition workshop. In a previous study, we evaluated objective data from student performance using an observed structured clinical exam (OSCE) station to assess the impact of the required nutrition workshop [12]. We planned to evaluate students regarding their nutrition counseling skills during an OSCE; however, due to the COVID-19 pandemic OSCEs were modified and not completed in person. 

## 5. Conclusions

The results of this study show that the introduction of interactive nutrition workshops in preclinical years likely increases students’ perceived knowledge of nutrition, comfort in completing a nutrition assessment, and confidence in advising patients about nutrition. These learned skills in nutrition counseling will help students to integrate nutrition counseling behaviors into their practice as future physicians and help them to prevent and manage disease and provide invaluable care to patients. We are hopeful that the results from our study will both motivate and empower other medical schools to increase the quality of nutrition education offered in their preclinical medical school curriculum. 

## Data Availability

The data presented in this study are available on request from the corresponding author.
